# Distinguishing hypertensive cardiomyopathy from cardiac amyloidosis in hypertensive patients with heart failure: a CMR study with histological confirmation

**DOI:** 10.1007/s10554-024-03262-0

**Published:** 2024-10-17

**Authors:** Katarzyna Elzbieta Gil, Vien Truong, Chuanfen Liu, Dalia Y. Ibrahim, Katarzyna Mikrut, Anjali Satoskar, Juliet Varghese, Rami Kahwash, Yuchi Han

**Affiliations:** 1 Division of Cardiovascular Medicine, The Ohio State University Wexner Medical Center, 452 W 10th Ave Columbus, Columbus, OH 43210 USA; 2 Department of Internal Medicine, Nazareth Hospital, Philadelphia, PA USA; 3 Department of Cardiology, Peking University People’s Hospital, Beijing, China; 4 Department of Pathology, University of Toledo Medical Center, Toledo, OH USA; 5 Advocate Heart Institute, Advocate Lutheran General Hospital, Chicago, IL USA; 6 Department of Pathology, The Ohio State University Wexner Medical Center, Columbus, OH USA; 7 Department of Biomedical Engineering, The Ohio State University, Columbus, OH USA

**Keywords:** Cardiovascular magnetic resonance imaging, Left ventricular hypertrophy, Hypertensive heart disease, Cardiac amyloidosis, Late gadolinium enhancement imaging, Extracellular volume fraction

## Abstract

**Purpose:**

Differentiation of the cause of left ventricular hypertrophy (LVH) is challenging in cases with co-existing hypertension. CMR offers assessment of diffuse myocardial abnormalities via T1 mapping with extracellular volume fraction (ECV) and macroscopic fibrosis via late gadolinium enhancement imaging (LGE). The goal of the study was to understand if CMR parameters can differentiate hypertensive cardiomyopathy (HC) from cardiac amyloidosis (CA) in patients with hypertension and heart failure, using endomyocardial biopsy (EMB) as the gold standard.

**Methods:**

We retrospectively analyzed patients with hypertension, LVH, and heart failure undergoing EMB due to uncertain diagnosis. CMR parameters including cine, LGE characteristics, T1 mapping, and ECV were analyzed.

**Results:**

A total of 34 patients were included (mean age 66.5 ± 10.7 years, 79.4% male). The final EMB-based diagnosis was HC (10, 29%), light chain (AL) CA (7, 21%), and transthyretin (ATTR) CA (17, 50%). There was a significant difference in subendocardial LGE (*p* = 0.03) and number of AHA segments with subendocardial LGE (*p* = 0.005). The subendocardial LGE pattern was most common in AL-CA (85.7%) and African American with HC (80%). ECV elevation (≥ 29%) was present in all patients with CA (AL-CA: 57.6 ± 5.2%, ATTR-CA: 59.1 ± 15.3%) and HC (37.3 ± 4.5%).

**Conclusions:**

Extensive subendocardial LGE pattern is not pathognomonic for CA but might also be present in African American patients with longstanding or poorly controlled HTN. The ECV elevation in HC with HF might be more significant than previously reported with an overlap of ECV values in HC and CA, particularly in younger African American patients.

**Graphical Abstract:**

**Figure:**
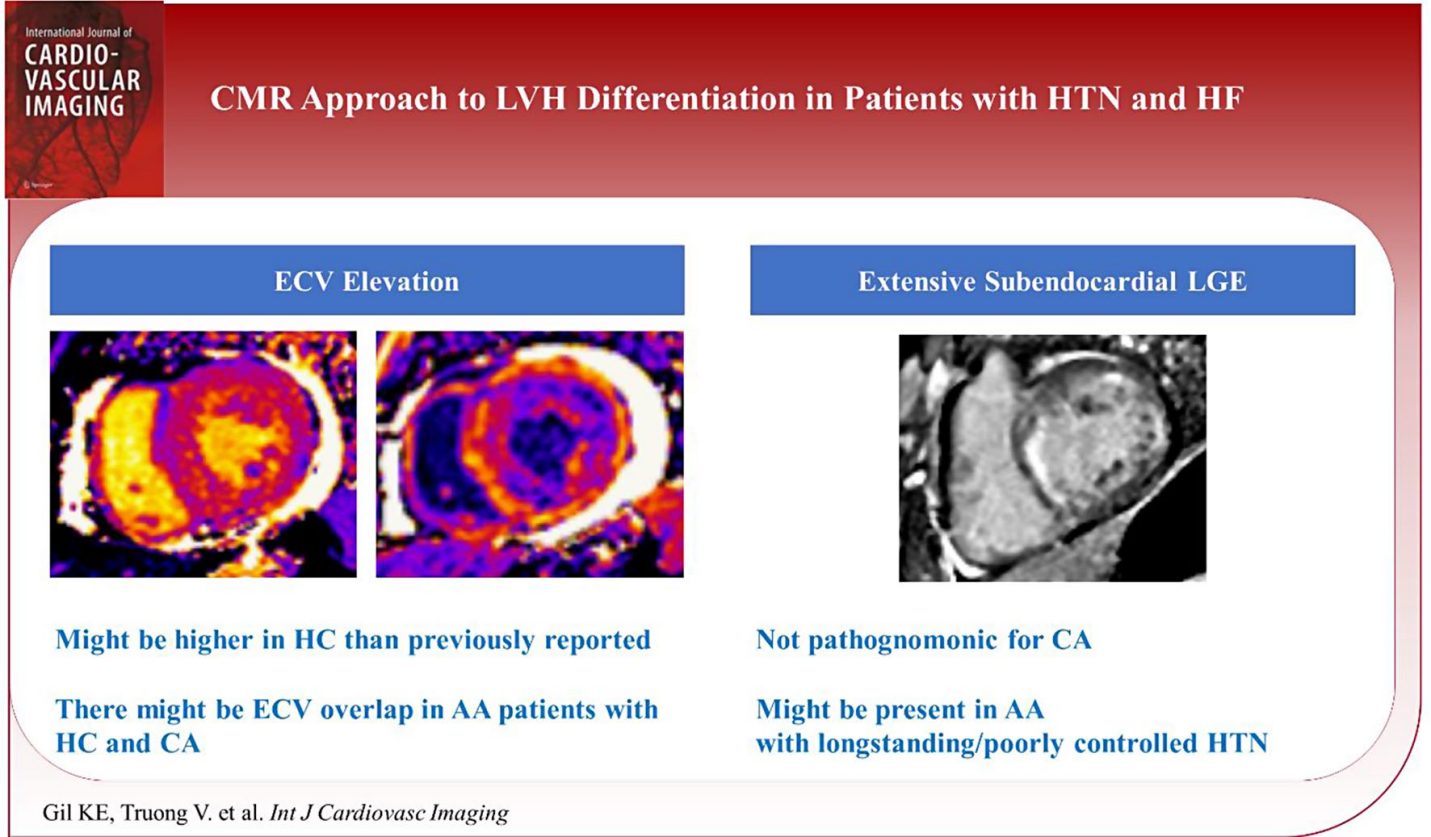
Abbreviations: CMR, cardiovascular magnetic resonance imaging; LVH, left ventricular hypertrophy; HTN, hypertension; HF, heart failure; ECV, extracellular volume fraction; HC, hypertensive heart disease; CA, cardiac amyloidosis; AA, African American; LGE, late gadolinium enhancement

**Supplementary Information:**

The online version contains supplementary material available at 10.1007/s10554-024-03262-0.

## Introduction

Left ventricular hypertrophy (LVH), defined as an increase in left ventricular mass or wall thickness, may be the compensatory response of cardiomyocytes to increased afterload, or the presentation of infiltrative diseases such as cardiac amyloidosis (CA) or other myocardial diseases such as hypertrophic cardiomyopathy (HCM) [1–3]. Cardiac magnetic resonance imaging (CMR) provides means of phenotyping the underlying pathology with unique opportunity for noninvasive assessment of LVH and myocardial characterization with diffuse extracellular expansion (T1 mapping and extracellular volume fraction [ECV]) and focal sequestration of gadolinium (late gadolinium enhancement [LGE]) [2–5].

In CA, the typical LGE pattern involves global subendocardial LGE in a non-coronary distribution [1, 2, 6–8]. However, LGE pattern might be variable in patients with CA - ranging from diffuse patchy subendocardial or transmural to rarely subepicardial pattern, often depending on the stage of presentation [2, 6, 9]. On the other hand, LGE in hypertensive cardiomyopathy (HC) is mostly non-specific, patchy, less prominent than in other LVH etiologies, and reported in approximately 50% of patients [1–3, 10, 11]. LGE in HCM is present in approximately 60-70% of cases and is typically midmyocardial and patchy, occurring predominantly in areas of maximum hypertrophy and at the RV insertion points [3, 5].

T1 mapping is an important discriminator of LVH etiologies with substantial pre-contrast T1 and ECV elevation (considered a surrogate marker for amyloid burden) as well as post-contrast T1 shortening in CA [1, 2, 12]. The native T1 signal is higher with AL than with ATTR CA, whereas the ECV is higher with ATTR CA [3, 6]. T1 and ECV typically range from normal to slightly elevated in HC and may also be increased in HCM [2, 3, 5, 13].

Despite best efforts, LVH differentiation might be challenging, especially at the early stage of CA and advanced stages of other LVH forms, particularly HC [6]. There are no clear-cut LGE patterns or clear cut-off values for T1 and ECV for individual LVH etiologies [14]. Definitive diagnosis cannot always be made without multiple diagnostic testing, longitudinal follow-up, and re-assessment to treatment response [2]. Whereas CMR may suggest the LVH etiology, the final diagnosis in most challenging cases might have to be based on endomyocardial biopsy (EMB) and genetic testing [1, 6, 8, 15].

The overall goal of the study is to determine if multiparametric CMR can help differentiate LVH in patients with heart failure (HF), hypertension (HTN), and features suspicious for CA, using histological evaluation as the gold standard for the final assignment of the study groups.

## Methods

### Patients

This is a retrospective cohort study assessing CMR parameters in non-ischemic patients with HTN, LVH, and HF undergoing histological evaluation due to uncertain diagnosis, despite extensive cardiac testing that included genetic testing and advanced cardiac imaging. Patients undergoing evaluation between September 22, 2008 and March 15, 2022, in two tertiary care centers (The Ohio State University Wexner Medical Center, Columbus, OH & University of Pennsylvania, Philadelphia, PA) were included in the analysis. The study was approved by the Institutional Review Boards who waived informed consent in the respective institutions. Inclusion criteria included age ≥ 18 years old, hypertension, LVH (defined as wall thickness ≥ 12 mm on CMR), and HF secondary to HC, ATTR CA, AL CA, or HCM [3, 4]. Prior myocardial infarction, myocarditis, septal ablation or myectomy were excluded. Patients with typical CMR findings of HC, HCM or CA who do not routinely undergo histological assessment were not included in this study. The final diagnosis was based on myocardial histology on EMB or transplant. Figure S1 demonstrates the decision-making process in that patient population.

Patients were classified into three groups based on the histological evaluation – HC, ATTR CA, and AL CA [16]. Additional subtyping based on histology was performed to differentiate AL from ATTR CA [15]. HC was defined as LVH associated with diastolic or systolic left ventricular (LV) dysfunction in patients with persistent systemic HTN, in the absence of other causes for increased afterload and a negative EMB result [4]. None of the histological studies performed in patients with HTN, LVH, and HF of uncertain etiology within the study period were positive for any other etiology such as HCM or Fabry disease. To ensure complete differential diagnosis, CMR findings of patients with established HCM diagnosis (defined as presence of LV wall thickness ≥ 15 mm not explained by loading conditions) confirmed on histological evaluation (of explanted heart or myocardium removed during septal myectomy) were analyzed for comparison with the main three study groups (HC, ATTR CA, AL CA). The electronic medical record was reviewed for demographic and clinical data as well as clinically performed cardiac testing including electrocardiography (ECG), Holter ECG stress testing, echocardiography, coronary computed tomography angiography, single photon emission computed tomography, positron emission tomography, technetium-99 m-pyrophosphate scintigraphy (PYP scan), left heart catheterization, endomyocardial biopsy (EMB), and genetic testing.

### **CMR** image acquisition & analysis

Clinical CMR images were acquired on three 1.5T scanners (MAGNETOM Avanto/Avanto fit/Sola, Siemens Healthineers, Erlangen, Germany) using standardized protocols including cine imaging, native and post-contrast T1 mapping, and LGE imaging [17].

Breath-held segmented bSSFP cine images were acquired in patients able to hold their breath in standard 2-, 3- and 4-chamber long axis and stack of short axis (SAX) views covering the entire LV. In cases of difficulties with breath-holding, free-breathing real-time cine images were acquired [18, 19].

T1 relaxation times were measured by the MOLLI (Modified Look-Locker Inversion Recovery) technique, using both prototype (WIP 448 and WIP 780B on Avanto systems) and commercially available sequences (MyoMaps on the Sola system) [20]. Native T1 maps were acquired either with a 3(3)3(3)5 (WIP 448) or with a 5(3 beat)3 acquisition scheme (WIP 780B and Myomaps) with the following acquisition parameters: field of view (240–400) mm x (320–450) mm, acquisition matrix size (192–256) x (108–170), reconstructed spatial resolution (1.4–2.1) x (1.4–2.1) mm, slice thickness 8 mm, flip angle 35º, bandwidth 930–1085 Hz/pixel, TI start time 100–111 ms and TI increment of 80 ms.

Different contrast agents including Gadavist (Bayer Healthcare Pharmaceuticals, 18 patients), Magnevist (Bayer HealthCare Pharmaceuticals, 4 patients), Multihance (Bracco Diagnostics, 4 patients), Dotarem (Guerbet LCC, 6 patients), and Prohance (Bracco Diagnostics, 2 patients) were administered clinically to obtain post-contrast T1 and LGE images during the study period. Contrast dose was based on weight at 0.15–0.2 mmol/kg.

Post-contrast T1 maps were acquired either with a 3(3)3(3)5 (WIP 448) or with a 4(1)3(1)2 acquisition scheme (WIP 780B and Myomaps) 14.5 min (IQR: 11–17) after injection of gadolinium-based contrast agent. The acquisition parameters were as follows: field of view (320–450) mm x (250–400) mm, acquisition matrix size (192–256) x (120–162), reconstructed spatial resolution (1.4–2.3) x (1.4–2.3) mm, slice thickness 8 mm, flip angle 35º, bandwidth 930–1085 Hz/pixel, TI start time 100 ms with increment in 80 ms.

Phase sensitive inversion recovery (PSIR) LGE images were acquired with a free-breathing motion-corrected and averaged single-shot inversion recovery prepared bSSFP method (MOCO LGE with 8 averages) in standard 2-, 3- and 4-chamber long axis and SAX views and/or with a free-breathing single-shot single-average inversion recovery prepared bSSFP method in a SAX stack covering the LV. Representative acquisition parameters were as follows: field of view (240–400) mm x (320–500) mm, acquisition matrix size (102–192) x (192–256), reconstructed spatial resolution (1.4–2.6) x (1.4–2.6) mm, slice thickness 8 mm, flip angle 40–55º, and bandwidth 975–1532 Hz/pixel.

CMR studies were anonymized and analyzed using SuiteHeart (Neosoft, LLC, Pewaukee, Wisconsin). LV volumes and LVEF were measured from SAX images that covered the LV. Native and post-contrast myocardial T1 values were measured within the septum on the mid SAX maps, excluding confluent LGE. Septal T1 was measured, in order to avoid measurement biases in quantification of ECV and myocardial fibrosis [21]. ECV was calculated using the standard formula based on T1 mapping and hematocrit obtained on the day of the CMR study [22]. Elevated ECV was defined as ≥ 29% and was based on institutionally established normative data (same threshold in both tertiary care centers).

The presence, pattern, and extent of LGE on long axis (2-, 3-, and 4-chamber views) and SAX images were assessed by two level 3 trained CMR readers blinded to clinical information. LGE patterns were described as follows: subendocardial, midwall, subepicardial, and transmural LGE. Presence of LGE in papillary muscles (PM) and base-apex gradient was also assessed [7, 8]. Extent of LGE was reported using the full-width half-maximum technique according to the American Heart Association [AHA] 17-segment model [23, 24]. To assess inter-observer variability, two level 3 CMR readers performed the analysis of T1 mapping and LGE using a random set of 10 patients. To assess the intra-observer variability, one of the readers performed the analysis of T1 mapping and LGE twice in the same set of 10 patients at least two weeks apart.

### Cardiac histology

Cardiac histology was based either on EMB or native myocardium examination in patients undergoing heart transplant. EMB consisted of 5 specimens obtained from the RV septal wall. Histologic sections were examined by standard hematoxylin and eosin, trichrome, iron, and Congo red stains. Immunohistochemical stains using antibodies directed against serum amyloid A, transthyretin, and kappa and lambda light chains were conducted in case of positive Congo red stain. If the CA classification could not be made, mass spectrometry was performed. Electron microscopy studies were performed depending on the histological results and pathologist’s assessment.

### Statistical analysis

Categorical variables are summarized using frequency (percentage), and comparison between groups was performed using the chi-square test or exact test. Continuous variables are expressed as mean ± standard deviation (SD) for normal distributions and median + interquartile range for non-normal distributions. The distribution of continuous variables was determined using skewness, kurtosis, visual inspection of the histogram, and QQ plot. T test or Mann–Whitney U test was used to compare differences between the two groups for normally and non-normally distributed variables respectively. Comparisons between three or more groups were made with a one-way analysis of variance (ANOVA). Given unequal sample sizes among groups, the Welch’s test was performed.

With regards to reproducibility assessment, intra- and inter-observer reproducibility for CMR parameters were analyzed using the Bland–Altman method, and intra-class correlation coefficients (ICC, two-way random, absolute agreement, and single measure) for continuous variables, and the Kappa value and proportion in agreement for categorical variables. For continuous variables, agreement was considered excellent when ICC > 0.74, good when ICC = 0.60–0.74, fair when ICC = 0.40–0.59, and poor when ICC < 0.4. For categorical variables, Kappa value was interpreted as follows: values ≤ 0 as no agreement and 0.01–0.20 as none to slight, 0.21–0.40 as fair, 0.41– 0.60 as moderate, 0.61–0.80 as substantial, and 0.81–1.00 as almost perfect agreement. A two-sided P-value of < 0.05 was considered statistically significant. A two-sided P-value of < 0.05 was considered statistically significant. The statistical analyses were performed using IBM SPSS Statistics for Windows, version 22.0 (IBM Corp., Armonk, N.Y., USA) and R software, version 4.0.3 (The R Foundation, Vienna, Austria).

## Results

### Baseline characteristics

A total of 34 patients were included in the analysis. 23 CMR studies were obtained on Avanto scanner, 2 on AvantoFit scanner, and 9 on Sola scanner. The mean age of the cohort was 66.5 ± 10.7 years, with 79.4% being male (Table 1). There were significant differences among the patients assigned to three study groups (HC, ATTR CA, and AL CA) in age (*p* < 0.001), sex (*p* = 0.02), race (*p* = 0.01), diastolic blood pressure (*p* = 0.02), cerebrovascular accident/transient ischemic attack (*p* = 0.04), ECG parameters (low voltage QRS complexes (*p* = 0.005), left ventricular hypertrophy (*p* = 0.005), and left ventricular stroke volume index (*p* = 0.04). There were no other significant differences in comorbidities, pharmacotherapy, LV wall thickness, LV and RV volumes or function. Detailed baseline and CMR characteristics are summarized in Tables S1-S3.

**Table 1:** Baseline patient characteristics of the whole study population of patients with hypertension and heart failure, as well as study groups defined by the final diagnosis of hypertensive cardiomyopathy, AL Amyloidosis, and ATTR Amyloidosis

Characteristic	Whole cohort (*n* = 34)	HC (*n* = 10)	AL Amyloidosis (*n* = 7)	ATTR Amyloidosis (*n* = 17)	*P* value
Age, mean (SD), y	66.5 ± 10.7	55.3 ± 8.9	68.0 ± 7.2	72.1 ± 8.1	< 0.001
Male, No. (%)	27 (79.4)	8 (80.0)	3 (42.9)	16 (94.1)	0.02
African American, No. (%)	19 (57.6)	9 (90.0)	1 (16.7)	9 (52.9)	0.01
BSA, mean (SD), m^2^	2.0 ± 0.2	2.1 ± 0.2	1.9 ± 0.2	2.1 ± 0.2	0.14
BMI, mean (SD), kg/m^2^	28.2 ± 5.4	29.9 ± 5.9	25.6 ± 5.3	28.2 ± 4.9	0.27
SBP, mean (SD), mmHg	126.9 ± 20.6	132.9 ± 25.7	110.7 ± 11.1	130.1 ± 17.6	0.06
DBP, mean (SD), mmHg	75.4 ± 12.6	76.7 ± 15.5	63.9 ± 4.7	79.4 ± 10.4	0.02
HR, mean (SD), beats/min	73.6 ± 12.0	70.0 ± 14.7	82.0 ± 11.2	72.4 ± 9.4	0.10
Hyperlipidemia	19 (55.9)	6 (60.0)	4 (57.1)	9 (52.9)	0.94
Diabetes mellitus	10 (29.4)	5 (50.0)	1 (14.3)	4 (23.5)	0.30
Chronic Kidney Disease	23 (67.6)	7 (70.0)	6 (85.7)	10 (58.8)	0.45
Atrial Arrhythmia	20 (58.8)	7 (70.0)	2 (28.6)	11 (64.7)	0.20
Coronary Artery Disease	19 (55.9)	5 (50.0)	3 (42.9)	11 (64.7)	0.67
Obstructive Coronary Disease	7 (35.0)	1 (16.7)	1 (33.3)	5 (45.5)	0.68
Cerebrovascular Accident/Transient Ischemic Attack	5 (14.7)	4 (40.0)	0 (0)	1 (5.9)	0.04
VT ablation	2 (6.1)	1 (11.0)	0 (0.0)	1 (5.9)	0.99
PVC/VT	8 (23.5)	2 (20.0)	2 (28.6)	4 (23.5)	0.99
AV block	9 (26.5)	1 (10)	2 (28.6)	6 (35.3)	0.35
*Pharmacotherapy*,* No. (%)*					
Antiplatelets	21 (61.8)	7 (70)	6 (85.7)	8 (47.1)	0.20
Statins	24 (70.6)	9 (90)	6 (85.7)	9 (52.9)	0.10
Betablockers	22 (64.7)	8 (80.0)	2 (28.6)	12 (70.6)	0.09
ACE-inhibitors	7 (20.6)	3 (30.0)	1 (14.3)	3 (17.6)	0.74
Angiotensin receptor blockers	9 (26.5)	2 (20.0)	1 (14.3)	6 (35.3)	0.68
Sacubitril/Valsartan	2 (5.9)	2 (20)	0 (0)	0 (0)	0.12
Antiarrhythmics	7 (20.6)	3 (30.0)	1 (14.3)	3 (17.6)	0.74
Diuretics	27 (79.4)	9 (90.0)	4 (57.1)	14 (82.4)	0.29
Mineralocorticoid Receptor Antagonists	14 (41.2)	4 (40.0)	2 (28.6)	8 (47.1)	0.82
*Ecg*,* No. (%)*					
Low Voltage QRS Complexes	12 (35.3)	1 (10.0)	6 (85.7)	5 (29.4)	0.005
Pseudo-infarct Pattern	5 (14.7)	0 (0.0)	1 (14.3)	4 (23.5)	0.38
Left Ventricular Hypertrophy	4 (11.8)	4 (40.0)	0 (0)	0 (0)	0.005
Deep Q Waves in Lateral/Inferior Leads	4 (11.8)	0 (0.0)	1 (14.3)	3 (17.6)	0.40
Apical HCM Pattern	0 (0.0)	0 (0.0)	0 (0.0)	0 (0.0)	NA
*Holter ecg*,* No. (%)*					
PVCs/nonsustained VT	14 (41.2)	3 (30.0)	4 (57.1)	7 (41.2)	0.53
Sustained VT	2 (5.9)	1 (10.0)	1 (14.3)	0 (0.0)	0.24
PYP Scan, No. (%)	11 (32.4)	6 (60)	0 (0)	5 (29.4)	0.04
Positive PYP Scan, No. (%)	4 (36.4)	0 (0)	NA	4 (80.0)	0.02
Genetic Testing, No. (%)	18 (52.9)	4 (40.0)	1 (14.3)	13 (76.5)	0.01
Positive Genetic Testing, No. (%)	9 (50.0)	1 (25.0)	0 (0.0)	8 (61.5)	0.29
Mutations	N/A	- TTR (Val142ile)	N/A	-TTR (Val142ile); -TTR (val122ile); 1-TTR (Thr60Ala); -TTR (Ile88Leu); -TTR (V30M)	N/A

HC indicates hypertensive cardiomyopathy; AL, light chain; ATTR, transthyretin; BSA, body surface area; BMI, body mass index; SBP, systolic blood pressure; DBP, diastolic blood pressure; HR, heart rate; VT, ventricular tachycardia; ecg, electrocardiography; PVC, premature ventricular complex; ACE, angiotensin-converting enzyme; PYP, technetium pyrophosphate scintigraphy; and TTR, transthyretin

### Characteristics of patients with HC

HC patients were predominantly male (8/10), African American (9/10) (Table S1) and significantly younger than CA patients (Table 1). Their medical history was remarkable for longstanding/poorly controlled HTN with chronic kidney disease/end-stage renal kidney disease (4/10); longstanding/poorly controlled HTN, end-stage renal kidney disease and TTR mutation (1/10); longstanding/poorly controlled HTN with multiple myeloma (1/10); longstanding HTN with CAD (1/10); short history of HTN with multiple myeloma (1/10); or short history of HTN with CAD (2/10). PYP scan was performed in 6/10 patients and was negative for TTR amyloidosis in all cases. Genetic testing was performed in 4/10 patients and negative in 3/10. CMR was suggestive of CA in 8/10 patients, cardiac sarcoidosis with lower concern for CA in 1/10 patients, and inconclusive in 1/10 patient. Subendocardial LGE in 1 patient with obstructive CAD did not correlate with the coronary distribution.

### Histological findings

**Fig. 1:**
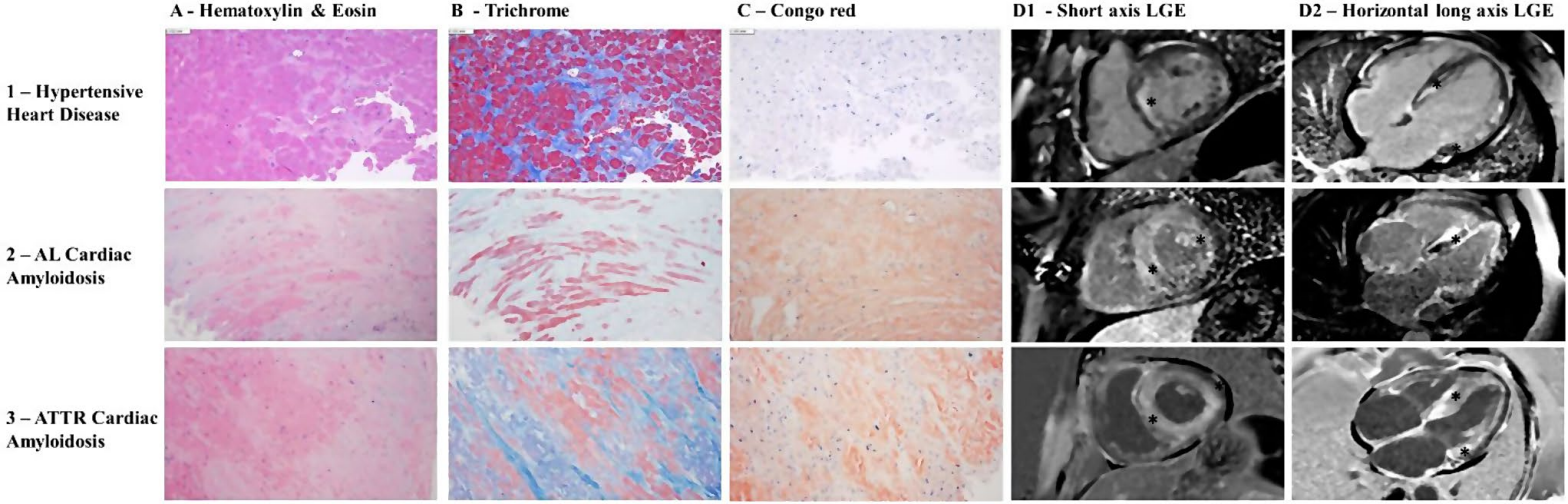
Histopathological workup of endomyocardial biopsy samples and late gadolinium enhancement on CMR images in study groups defined by the final diagnosis of hypertensive cardiomyopathy, AL Amyloidosis, and ATTR Amyloidosis. Panel 1: Cardiomyocyte hypertrophy (Panel 1 A), mild interstitial fibrosis (Panel 1 A), and no evidence of amyloid deposits (Panel 1 C) in a patient with hypertensive cardiomyopathy. Midmyocardial and subendocardial late gadolinium enhancement in the left ventricle (Panel 1D1-D2, asterisks). Panel 2: Deposition of nonstructural substance in the myocardial interstitium (Panel 2 A), interstitial fibrosis (Panel 2B), and amyloid deposits (Panel 2 C) in a patient with AL amyloidosis. AL amyloidosis further confirmed by positive immunoperoxidase staining for amyloid A in the amyloid deposits. Subendocardial late gadolinium enhancement in the left ventricle with papillary muscles involvement (Panel 2D1-D2, asterisks). Panel 3: Deposition of nonstructural substance in the myocardial interstitium (Panel 3 A), interstitial fibrosis (Panel 3B), and amyloid deposits (Panel 3 C) in a patient with ATTR amyloidosis. TTR amyloidosis further confirmed by positive immunoperoxidase stain for prealbumin/transthyretin, negative for kappa and lambda light chains and mass spectrometry. Extensive midmyocardial and near transmural late gadolinium enhancement in the left ventricle with papillary muscles involvement (Panel 3D1-D2, asterisks)

33 patients underwent EMB, and the remaining 1 patient had a heart transplant. The median time between histological evaluation and CMR was 26 days (IQR: 4–78 days). The histological results were positive for AL CA in 7 patients, ATTR CA in 17 patients, HC in the remaining 10 patients (Fig. 1).

**Fig. 2:**
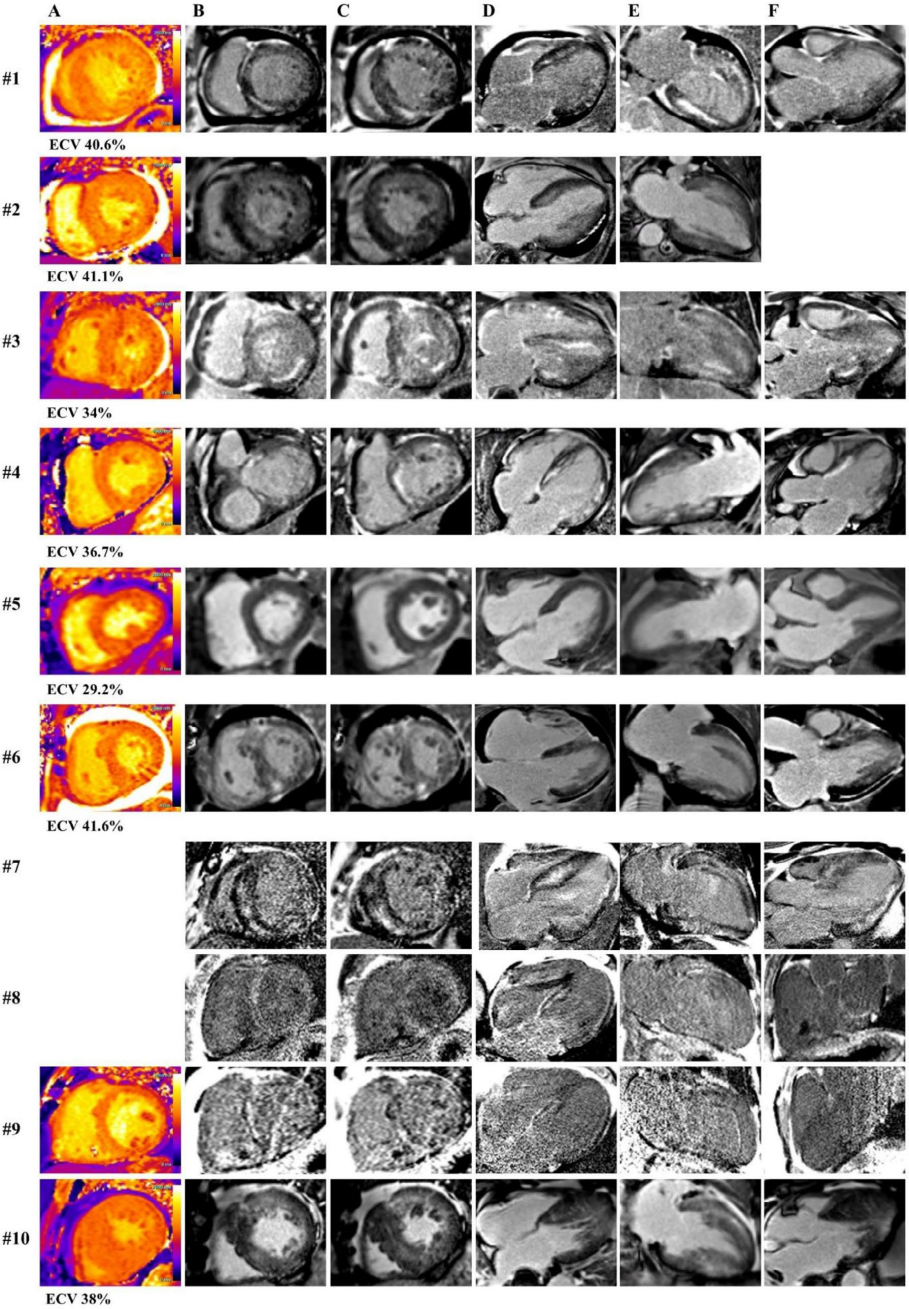
T1 mapping (**A**) with calculated extracellular volume fraction (ECV) and late gadolinium enhancement imaging (**B**-**F**) in patients with hypertensive cardiomyopathy (#1–10). A – mid short axis, B – base short axis, C – mid short axis, D – four chamber view, E – two chamber view, F – 3 chamber view

CA was ruled out based on EMB in 8 patients after a CMR study highly suggestive of CA (Table S1). All 8 patients were finally diagnosed with HC. 8/8 patients were African American with extensive LGE on CMR imaging, and negative Congo red staining on EMB. The predominant LGE patterns were subendocardial and transmural (Fig. 2).

### Myocardial nulling and LGE patterns

**Fig. 3:**
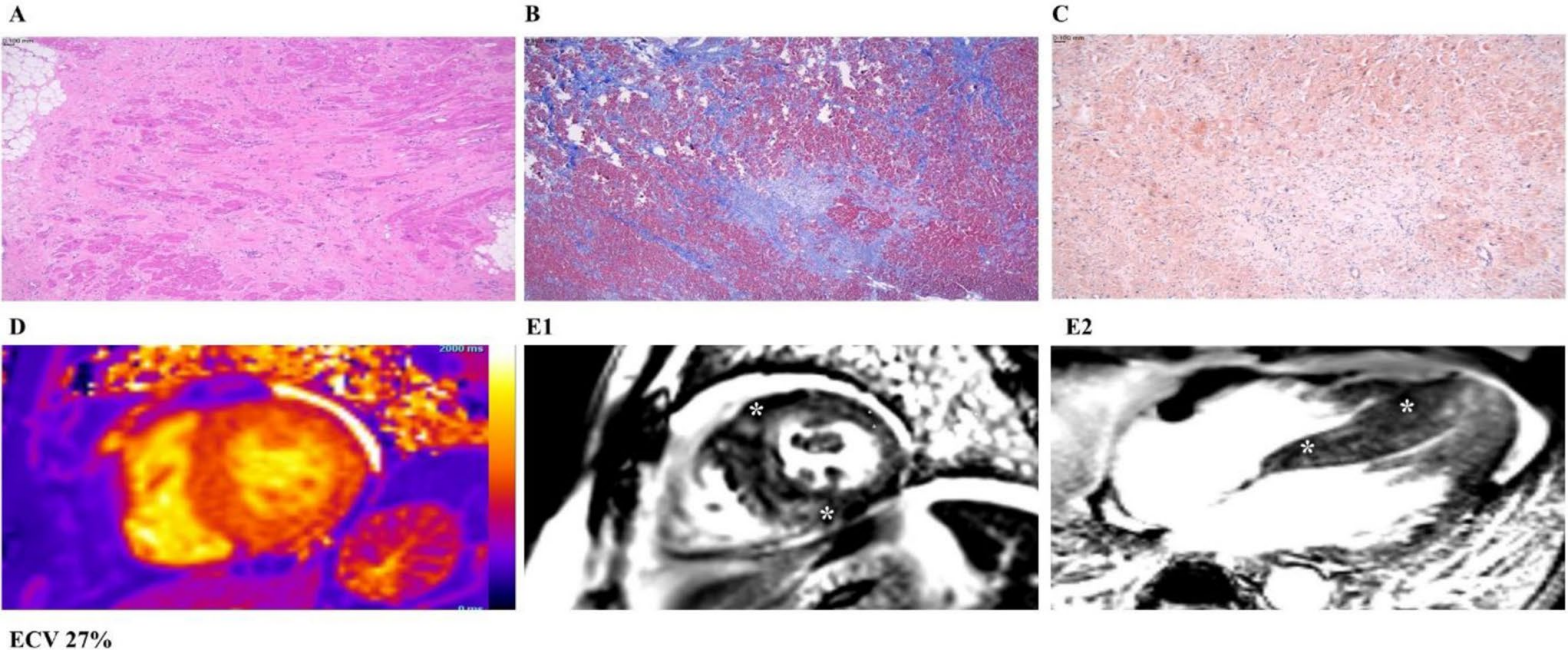
Histopathological evaluation of explanted heart, along with T1 mapping and late gadolinium enhancement on CMR images, in a patient with established diagnosis of hypertrophic cardiomyopathy. Myocyte hypertrophy (Panel A, Hematoxylin & Eosin) with areas of fibrosis (Panel B, Trichrome), and no evidence of amyloid deposits (Panel C, Congo Red). Normal T1 and ECV values. Midmyocardial late gadolinium enhancement in the septum and RV insertion points (Panel E1- short axis; E2 – horizontal long axis LGE, asterisks)

Since coincident myocardial and blood pool nulling was present in 3/10 HC patients, and not all patients with CA demonstrated abnormal myocardial nulling (17/24), abnormal myocardial nulling and poor contrast between myocardium and blood pool were not sufficient to distinguish advanced HC and CA. LGE was present in all 34 (100%) patients. The most common pattern was midwall LGE (82.4%), subendocardial (58.8%), transmural (35.3%), and subepicardial (5.9%) pattern (Table 2, Supplement, Fig. 2). The median number of involved AHA segments was 17 with a mean LGE extent of 38.1% (Table 2, Supplement, Fig. 3). There was significant difference in subendocardial LGE (*p* = 0.03) including subendocardial LGE in the basal LV segments (*p* = 0.01), number of AHA segments with subendocardial LGE (*p* = 0.005), and LGE in PM (*p* = 0.004) with no difference in LGE extent, transmural LGE, or number of AHA segments with LGE (Table 2).

**Table 2:** CMR parameters of the whole study population of patients with hypertension and heart failure, as well as study groups defined by the final diagnosis of hypertensive cardiomyopathy, AL Amyloidosis, and ATTR Amyloidosis

Characteristic	Whole cohort (*n* = 34)	HC (*n* = 10)	AL Amyloidosis (*n* = 7)	ATTR Amyloidosis (*n* = 17)	*P* value
*CMR parameters*,* mean (SD)*					
Maximum Wall Thickness, cm	1.92 ± 0.45	1.75 ± 0.59	1.86 ± 0.47	2.05 ± 0.32	0.22
Septum Thickness, cm	1.65 ± 0.34	1.53 ± 0.40	1.60 ± 0.35	1.74 ± 0.29	0.28
LVEDVI, mean (SD), ml/m^2^	89.6 ± 25.5	103.5 ± 38.4	77.8 ± 13.7	88.0 ± 19.6	0.14
LV mass /LVEDV (SD), g/ml	1.15 ± 0.27	1.04 ± 0.35	1.19 ± 0.33	1.19 ± 0.18	0.36
LVESVI, mean (SD), ml/m^2^	53.3 ± 22.3	59.1 ± 34.9	44.5 ± 9.9	54.3 ± 18.6	0.45
LVEF, mean (SD), %	42.1 ± 10.4	45.2 ± 11.4	42.9 ± 8.3	39.9 ± 10.7	0.45
LVSVI, mean (SD), ml/m^2^	36.4 ± 10.4	44.4 ± 12.8	33.3 ± 8.7	34.0 ± 8.3	0.04
LV mass index, mean (SD), g/m^2^	102.4 ± 30.5	105.5 ± 37.5	91.0 ± 21.3	105.2 ± 29.7	0.56
RVEDVI, mean (SD), ml/m^2^	90.4 ± 25.4	86.9 ± 27.2	78.3 ± 12.2	97.0 ± 27.5	0.24
RVESVI, mean (SD), ml/m^2^	53.3 ± 21.7	45.8 ± 17.9	44.9 ± 7.6	60.3 ± 25.3	0.15
RVEF, mean (SD), %	42.1 ± 9.4	45.8 ± 8.2	42.4 ± 5.7	39.8 ± 10.9	0.28
RVSVI, mean (SD), ml/m^2^	37.1 ± 9.9	41.0 ± 11.6	33.4 ± 7.4	36.8 ± 9.8	0.33
*LGE analysis*					
LGE presence, No. (%)	34 (100)	10 (100)	7 (100)	17 (100)	NA
Min 1 segment with subendocardial LGE, No. (%)	20 (58.8)	8 (80)	6 (85.7)	6 (35.3)	0.03
Min 1 segment with midmyocardial LGE, No. (%)	28 (82.4)	9 (90.0)	4 (57.1)	15 (88.2)	0.21
Min 1 segment with subepicardial LGE, No. (%)	2 (5.9)	1 (10.0)	0 (0.0)	1 (5.9)	0.99
Min 1 segment with transmural LGE, No. (%)	12 (35.3)	1 (10.0)	4 (57.1)	7 (41.2)	0.13
Base/Apex Visual Gradient	6 (17.6)	1 (10.0)	1 (14.3)	4 (23.5)	0.84
LGE in Papillary Muscles	24 (70.6)	3 (30.0)	6 (85.7)	15 (88.2)	0.004
LGE basal subendocardial, No. (%)	16 (47.1)	6 (60.0)	6 (85.7)	4 (23.5)	0.01
AHA segments with LGE, median (IQR)	17.0 (17.0–17.0)	15.5 (13.8–17.0)	17.0 (17.0–17.0)	17.0 (12.5–17.0)	0.34
AHA segments with subendocardial LGE, median (IQR)	2.0 (0.0-5.5)	4.5 (0.8–6.3)	8.0 (4.0–15.0)	0.0 (0.0-2.5)	0.005
AHA segments with midmyocardial LGE, median (IQR)	9.0 (2.8–14.0)	9.0 (4.5–14.5)	2.0 (0.0–9.0)	12.0 (4.0–15.0)	0.21
AHA segments with transmural LGE, median (IQR)	0.0 (0.0-3.5)	0.0 (0.0-0.3)	1.0 (0.0–5.0)	0.0 (0.0-5.5)	0.35
AHA segments with subendocardial and transmural LGE, median (IQR)	4.0 (0.0-14.3)	4.5 (0.8–7.3)	15.0 (4.0–17.0)	2.0 (0.0-9.5)	0.07
LGE extent, mean (SD)	38.1 (31.1–44.9)	33.9 (30.8–40.7)	34.6 (31.1–44.0)	41.8 (32.3–48.3)	0.28
*T1 mapping analysis*					
Native myocardial T1 time, mean (SD), ms^**†**^	1104.0 ± 62.1	1110.1 ± 42.1	1081.1 ± 72.9	1110.9 ± 66.7	0.56
Post-contrast myocardial T1 time, mean (SD), ms^**†**^	327.0 ± 74.4	394.5 ± 43.8	339.7 ± 56.7	287.8 ± 68.8	0.001
ECV, mean (SD)^**#**^, %	53.7 ± 14.7	37.3 ± 4.5	57.6 ± 5.2	59.1 ± 15.3	0.001
ECV ≥ 29%^**#**^, No. (%)	30 (100)	7 (100)	7 (100)	16 (100)	

HC indicates hypertensive cardiomyopathy; AL, light chain; ATTR, transthyretin; CMR, cardiovascular magnetic resonance imaging; LVEDVI, left ventricular end-diastolic volume index; LVESVI, left ventricular end-systolic volume index; LVEF, left ventricular ejection fraction; LVSVI, right ventricular stroke volume index; RVEDVI, right ventricular end-diastolic volume index; RVESVI, right ventricular end-systolic volume index; RVEF, right ventricular ejection fraction; RVSVI, right ventricular stroke volume index; LGE, late gadolinium enhancement; AHA, American Heart Association; and ECV, extracellular volume fraction

The subendocardial LGE pattern was most common in patients with AL CA (85.7%) and HC (80%), whereas LGE in PM in AL CA (85.7%) and ATTR CA (88.2%).

### T1 mapping

The median time from injection to post T1 acquisition was 14.5 min (IQR: 11–17). There were significant differences between groups in post-contrast myocardial T1 (*p* = 0.001) and ECV values (*p* = 0.001; Table 2) with the lowest T1 and highest ECV values in patients with AL CA (T1: 339.7 ± 56.7 ms, ECV: 57.6 ± 5.2%) and ATTR-CA (T1: 287.8 ± 68.8 ms, ECV: 59.1 ± 15.3). ECV elevation (≥ 29%) was observed in all patients with CA and HC (AL-CA: 100%; ATTR CA: 100%; HC: 100% of patients with T1 mapping performed).

### CMR characteristics of patients with established HCM diagnosis

CMR studies of 6 patients with established HCM diagnosis, who underwent heart transplant or septal myectomy during the study period, were compared with the other LVH groups (HC, ATTR CA, AL CA). There were no significant differences in LV wall thickness (max thickness 2.1 ± 0.3 cm, *p* = 0.31), volume (LV end diastolic volume index 104.1 ± 27.2 ml/m2, *p* = 0.12), or function (LV ejection fraction 42.3 ± 18.1%, *p* = 0.51) between HCM and other LVH etiologies. LGE was present in all HCM patients (100%) with midmyocardial LGE pattern being most predominant (100%), followed by RVIP (83.3%), subendocardial (16.7%), and subepicardial LGE (16.7%)(Fig. 3). Transmural LGE was not present in any HCM patients (0%). LGE was less significant with the median number of involved AHA segments of 10 (IQR: 5–11, *p* = 0.002) and a mean LGE extent of 5.8% (2.2–21.3%, *p* = 0.004). LGE was not present in the papillary muscles (0%). T1 mapping was performed in 50% of HCM patients with T1 measured at 1064.3 ± 85.6 ms, and ECV at 26.1 ± 2% (*p* < 0.001). ECV elevation, on the contrary to other LVH groups, was not demonstrated in any HCM patients (0%, *p* < 0.001).

### Intra-observer and inter-observer reproducibility

The intra-observer and inter-observer reproducibility was excellent for T1 mapping, ECV, and LGE parameters (Supplement, Table S2).

## Discussion

We investigated quantitative T1 and ECV values, the LGE patterns and their role in LVH etiology differentiation in non-ischemic patients with HTN and HF using histology as the gold standard. Despite the limited number of patients, our findings concerning LGE images should be taken as a preliminary indicator that the presence of extensive subendocardial LGE pattern with ECV elevation is not always indicative of CA. Patients with an advanced stage of HC, particularly African Americans, might also present with extensive subendocardial LGE in a non-coronary distribution. However, these HC patients tend to be younger (mean age in the 50’s versus 70’s for TTR) with evidence of LVH on ECG. The number of segments with subendocardial LGE appears to be lower in ATTR CA than in AL CA.

The subendocardial LGE pattern in a non-coronary distribution may occur not only in CA but also in other myocardial diseases such as sarcoidosis or HCM, due to extensive fibrosis [5, 7, 15, 25]. Furthermore, since CMR assessment of the subendocardium might be limited by partial volume effect of the blood tissue interface, subendocardial LGE might be underappreciated [26]. Most patients with extensive fibrosis and HC in our study were African Americans. Excess end organ damage in African Americans with HTN is associated with genetically and/or environmentally induced amplification of profibrotic mechanisms, such as the renin angiotensin system and transforming growth factor beta [27]. Since interstitial fibrosis in African Americans patients with hypertensive nephrosclerosis has been proven to be more significant than in Caucasians, it seems probable that myocardial fibrosis in African Americans patients with HC might follow the same pattern [27]. Higher LV afterload in some African Americans leads to eccentric remodeling, cardiomyocyte death, and replacement fibrosis as opposed to concentric remodeling with reactive fibrosis in Caucasians [28, 29].

Diffuse subendocardial LGE has also been previously described in patients with multiple myeloma without evidence of systemic amyloidosis, seen also in two of our HC patients [30]. The mechanism of myocardial injury is unclear, but toxicity of the light chains might be a contributor [30].

Sampling error of the EMB resulting in erroneous assignment to the HC group of patients with CMR suggestive of CA cannot be excluded [8]. EMB assesses only the subendocardium of the RV, and amyloid deposition can be uneven, hence at least five biopsy specimens were obtained in every patient [16, 31]. Additionally, EMB was performed twice in two of the HC patients several years apart and remained negative. Absence of CA was further confirmed by a negative PYP scan in 6/8 patients with CMR suggestive of CA, and negative blood work-up for AL CA. Non-diagnostic myocardial tissue mapping, on the other hand, could potentially explain one false negative CMR for CA [8, 16].

Patients with CA are more likely to demonstrate LGE in the PM, however it is not pathognomonic and has been observed in a variety of conditions including HC, HCM, and mitral valve prolapse [32, 33]. In our cohort, PM LGE was present in CA and significantly less often in HC. PM LGE is most often focal and confined to the proximal PM portions or diffusely patchy [32]. The relatively high percentage of patients with PM LGE in our cohort might be partially explained by the inclusion of patients with advanced disease.

Postcontrast myocardial T1 time is inversely correlated with the presence of diffuse fibrosis on EMB in a population with a broad spectrum of cardiomyopathies [34]. However, significant post-contrast T1 shortening has not been previously described in HC. Our findings regarding ECV elevation in CA agree with already published data [2, 35]. The ECV elevation in HC, on the other hand, was more significant than previously reported with an overlap of ECV values in HC and CA [5, 13, 35, 36]. The population of the prior studies, however, comprised of patients without HF [5, 13, 36]. Since ECV has been shown to closely reflect the degree of diffuse myocardial fibrosis, higher values might be expected in patients with more significant LVH and HF [36]. In our study cohort, a negative PYP scan and ECV of less than 40% were highly suggestive but not definitive for HC. Patients with established HCM, however, may potentially be identified by normal ECV in areas not affected by LGE. While our study provides additional information about the role of native T1 and ECV in differentiating HC and CA, the findings in individual patients should be interpreted in the context of clinical findings and other cardiovascular tests.

It is worth mentioning that PYP scan did not confirm ATTR CA in 1 patient with CMR suggestive of advanced form of CA. This has been rarely reported in the literature (only in Phe64Leu and Val122Ile mutations) and further emphasizes the importance of multimodality imaging in patients with LVH [37, 38].

### Limitations

This is a small retrospective analysis, and as such, the sensitivity and specificity of using native T1 and ECV as a definitive differentiator between CA and HC should be assessed in a larger multicenter study. Second, our definition of HC relied on the presence of increased wall thickness and history of HTN. This approach, which was also recently applied by Neisius and colleagues, was chosen to adjust for the major HCM diagnostic criterion based on LV wall thickness [14].

The study spanned a time period where technical optimization and standardization efforts for quantitative parametric mapping techniques in CMR practice were still underway. As such, the use of different image acquisition protocols over time may have contributed to greater variability in the measured T1 values across the population. Nevertheless, the findings regarding T1 and ECV values appear consistent and substantial, and do not seem to be solely dependent on the variability in acquisition parameters.

## Conclusions

The differential diagnosis of LVH in patients with HF and HTN remains challenging. The results of this retrospective analysis suggest that ECV elevation in HC with HF might be more significant than previously reported in a cohort of predominantly African American patients with poorly controlled HTN. LVH differentiation seems most complicated in advanced HC and at an early stage of CA, since LGE pattern and T1 mapping abnormalities overlap. Extensive subendocardial LGE pattern is not pathognomonic for CA but might also be present in African American patients with longstanding or poorly controlled HC with end organ damage. Among African American subjects, where both hereditary TTR and HC can be prevalent, careful examination of clinical (age being an important differentiator) and imaging data is required.

## Electronic supplementary material

Below is the link to the electronic supplementary material.

Supplementary Material 1

## Data Availability

Data is provided within the manuscript or supplementary information files. Additional data generated or analyzed during the study are available from the corresponding author by request.
