# Supplementary material for: Distinguishing hypertensive cardiomyopathy from cardiac amyloidosis in hypertensive patients with heart failure: a CMR study with histological confirmation
Source: Int J Cardiovasc Imaging. 2024 Oct 17;40(12):2559–70. doi: 10.1007/s10554-024-03262-0 (PMC11618216; doi:10.1007/s10554-024-03262-0)
Supplement: Supplementary file 1 — Supplementary Material 1 [file 10554_2024_3262_MOESM1_ESM.docx]

**Supplemental Material:**

Table S1-S4

Figures S1-S4

**Table S1.** Detailed Baseline (Table S1A) and CMR (Table S1B) Characteristics of Patients with Hypertensive Cardiomyopathy.

| **A.** Baseline Characteristics. | | | | | | | | | | | | | | | | | | | | | | | |
| --- | --- | --- | --- | --- | --- | --- | --- | --- | --- | --- | --- | --- | --- | --- | --- | --- | --- | --- | --- | --- | --- | --- | --- |
| Pt# | Gender (male – 1) | Race (AA – 1) | Age | | Death | | HTN (1-present; 2- poorly controlled; 3 – longstanding) | | HTN duration | | | DM | | 1 – CKD; 2- ESRD | | CAD | | HF | | Atrial arrhythmia | MM | | ACE-I/ARB/ARNI |
| 1 | 1 | 1 | 57 | | 0 | | 1,2,3 | | At least 24 yrs | | | 1 | | 1,2 | | 1 | | 1 | | 1 | 0 | | 1 |
| 2 | 1 | 1 | 65 | | 0 | | 1,2,3 | | At least 14 yrs | | | 1 | | 1,2 | | 1 | | 1 | | 1 | 0 | | 1 |
| 3 | 1 | 1 | 40 | | 0 | | 1,3 | | At least 6 yrs | | | 0 | | 1,2 | | 0 | | 1 | | 1 | 0 | | 1 |
| 4 | 1 | 0 | 56 | | 0 | | 1 | | At least 4 yrs | | | 1 | | 0 | | 1 | | 1 | | 0 | 0 | | 1 |
| 5 | 0 | 1 | 62 | | 0 | | 1 | | 2 yrs | | | 1 | | 1 | | 1 | | 1 | | 0 | 1 | | 1 |
| 6 | 1 | 1 | 55 | | 1 | | 1,2,3 | | 19 yrs | | | 0 | | 1 | | 0 | | 1 | | 1 | 1 | | 1 |
| 7 | 1 | 1 | 42 | | 0 | | 1,3 | | 22 years | | | 0 | | 1 | | 0 | | 1 | | 1 | 0 | | 1 |
| 8 | 0 | 1 | 79 | | 1 | | 1,3 | | 10 years | | | 0 | | 0 | | 1 | | 1 | | 0 | 0 | | 1 |
| 9 | 1 | 1 | 57 | | 0 | | 1 | | 6 years | | | 1 | | 0 | | 0 | | 1 | | 1 | 0 | | 1 |
| 10 | 1 | 1 | 64 | | 0 | | 1,2 | | At least 4 yrs | | | 0 | | 1 | | 0 | | 1 | | 1 | 0 | | 0 |
| **B.** Diagnostic characteristics. | | | | | | | | | | | | | | | | | | | | | | | |
| Pt# | Ischemic evaluation (1- done)/obstructive CAD (1 – positive) | EMB result (1- myocyte hypertrophy; 2-inflammation; 3-interstitial fibrosis; 4-“+” CA stains); 5 – EMB performed twice  /EMB date | | PYP scan (1- done, 2 – negative) | | Genetic testing (1 - done, 2 – positive) | | CMR result/  CMR date | | Max  Thickness (cm) | LVEDVI (ml/m2) | | LVEF (%) | | ECV | | LGE in the PM | | Number of segments  with subendocardial LGE | | Total number of LGE segments | LGE extent | |
| 1 | 1/0 | 1,3,5/  2006, 2018 | | 1,2 | | 0 | | Suggestive of CA/  2018 | | 1.7 | 142 | | 38 | | 40.6 | | 1 | | 2 | | 17 | 37.1 | |
| 2 | 1/0 | 1,3/  2021 | | 1,2 | | 0 | | Suggestive of CA/  2021 | | 2.2 | 129 | | 52 | | 41.1 | | 0 | | 5 | | 15 | 30.8 | |
| 3 | 1/0 | 1,3/2016 | | 1,2 | | 1,2 (TTR c.424G>A  mutation) | | Suggestive of CA/  2019 | | 1.7 | 94 | | 57 | | 34.0 | | 1 | | 5 | | 14 | 31.2 | |
| 4 | 1/1 | 1,3/  2020 | | 0 | | 0 | | CMR suggestive of sarcoidosis, lower concern for CA/  2020 | | 1.2 | 113 | | 40 | | 36.7 | | 0 | | 4 | | 13 | 36.6 | |
| 5 | 1/0 | 1,3/  2020 | | 0 | | 0 | | Multiple myeloma, CMR with nonischemic LGE  /2020 | | 1.2 | 54 | | 58 | | 29.2 | | 0 | | 0 | | 5 | 0 | |
| 6 | 0/0 | 1,2,3,5/  2016, 2021 | | 1,2 | | 1 | | Suggestive of CA/  2021 | | 1.3 | 79 | | 53 | | 41.6 | | 0 | | 5 | | 14 | 30.8 | |
| 7 | 1/0 | 1,3/  2018 | | 0 | | 0 | | Suggestive of CA/  2018 | | 2.8 | 158 | | 21 | | No T1 mapping | | 0 | | 1 | | 17 | 38.8 | |
| 8 | 1/0 | 1,3/  2012 | | 0 | | 1 | | Suggestive of CA/  2011 | | 1.4 | 140 | | 39 | | No T1 mapping | | 0 | | 10 | | 17 | 46.5 | |
| 9 | 1/0 | 1,3/  2019 | | 1,2 | | 1,2 (variant of unknown significance) | | Suggestive of CA/  2019 | | 1.4 | 94 | | 42 | | No HCT | | 1 | | 13 | | 17 | 50.7 | |
| 10 | 1/0 | 1,3/  2022 | | 1,2 | | 1 | | Suggestive of CA/  2022 | | 2.6 | 59 | | 52 | | 38 | | 0 | | 0 | | 16 | 31 | |

AA indicates African American; HTN, hypertension; DM, diabetes mellitus; CKD, chronic kidney disease; CAD, coronary artery disease; HF, heart failure; MM, multiple myeloma; ACE-I, angiotensin converting enzyme inhibitors; ARB, angiotensin receptor blockers; ARNI, angiotensin receptor neprilysin inhibitor; CAD, coronary artery disease; EMB, endomyocardial biopsy; CA, cardiac amyloidosis; PYP, Technetium Pyrophosphate Scintigraphy; CMR, cardiovascular magnetic resonance; LVEDVI, left ventricular end diastolic volume index; LVEF, left ventricular ejection fraction; ECV, extracellular volume fraction; LGE, late gadolinium enhancement imaging; and TTR, transthyretin.

**Table S2.** Detailed Baseline (Table S2A) and CMR (Table S2B) Characteristics of Patients with AL Amyloidosis.

| **A.** Baseline Characteristics. | | | | | | | | | | | | | | | | | | | | | | |
| --- | --- | --- | --- | --- | --- | --- | --- | --- | --- | --- | --- | --- | --- | --- | --- | --- | --- | --- | --- | --- | --- | --- |
| Pt# | Gender (male – 1) | Race (AA – 1) | Age | | Death | | HTN (1-present; 2- poorly controlled; 3 – longstanding) | | HTN duration | | DM | 1 – CKD; 2- ESRD | | CAD | | HF | | Atrial arrhythmia | | MM | | ACE-I/ARB/ARNI |
| 1 | 0 | 0 | 60 | | 1 | | 1 | | At least 4 years | | 0 | 1 | | 1 | | 1 | | 0 | | 0 | | 0 |
| 2 | 1 | 0 | 77 | | 0 | | 1, 3 | | At least 10 years | | 0 | 0 | | 0 | | 1 | | 0 | | 0 | | 0 |
| 3 | 0 | 0 | 78 | | 1 | | 1 | | At least 3 years | | 0 | 1 | | 0 | | 1 | | 0 | | 0 | | 0 |
| 4 | 1 | 0 | 61 | | 1 | | 1,3 | | At least 15 years | | 0 | 1 | | 1 | | 1 | | 1 | | 0 | | 1 |
| 5 | 1 | 1 | 67 | | 1 | | 1 | | At least 3 years | | 0 | 1 | | 1 | | 1 | | 0 | | 0 | | 1 |
| 6 | 0 | 0 | 69 | | 1 | | 1 | | At least 3 years | | 1 | 1 | | 1 | | 1 | | 0 | | 1 | | 0 |
| 7 | 0 | 0 | 64 | | 1 | | 1 | | Several years | | 0 | 1 | | 0 | | 1 | | 1 | | 0 | | 0 |
| **B.** Diagnostic characteristics. | | | | | | | | | | | | | | | | | | | | | | |
| Pt# | Ischemic evaluation (1- done)/obstructive CAD (1 – positive) | EMB result (1- myocyte hypertrophy; 2-inflammation; 3-interstitial fibrosis; 4-“+” CA stains); 5 – EMB performed twice/  EMB date | | PYP scan (1- done, 2 – negative) | | Genetic testing (1 - done, 2 – positive) | | CMR result/  CMRdate | | Max  Thickness (cm) | LVEDVI (ml/m2) | LVEF (%) | ECV | | LGE in the PM | | Number of segments  with subendocardial LGE | | Total number of LGE segments | | LGE extent | |
| 1 | 1/0 | 3,4/  2012 | | 0 | | 0 | | Suggestive of CA/  2012 | | 1.3 | 80 | 55 | 47.7 | | 0 | | 4 | | 11 | | 41.5 | |
| 2 | 1/0 | 4/  2019 | | 0 | | 0 | | Suggestive of CA/  2019 | | 2.0 | 92 | 43 | 62.2 | | 1 | | 0 | | 17 | | 51.6 | |
| 3 | 1/0 | 4/  2019 | | 0 | | 0 | | Suggestive of CA/  2019 | | 1.5 | 53 | 41 | 58 | | 1 | | 15 | | 17 | | 34.6 | |
| 4 | 1/1 | 3, 4/  2019 | | 0 | | 0 | | Suggestive of CA/  2019 | | 2.6 | 68 | 33 | 62.6 | | 1 | | 15 | | 17 | | 44 | |
| 5 | 1/0 | 4/  2018 | | 0 | | 0 | | Suggestive of CA/  2017 | | 2.3 | 77 | 52 | 59.3 | | 1 | | 7 | | 17 | | 34 | |
| 6 | 1/0 | 3, 4/  2020 | | 0 | | 0 | | Suggestive of CA/  2020 | | 1.6 | 86 | 42 | 53.9 | | 1 | | 8 | | 17 | | 31.1 | |
| 7 | 1/0 | 4/  2019 | | 0 | | 1 | | Suggestive of CA/  2019 | | 1.7 | 89 | 34 | 59.6 | | 1 | | 12 | | 17 | | 22.9 | |

AL indicates amyloid light chain; AA, African American; HTN, hypertension; DM, diabetes mellitus; CKD, chronic kidney disease; CAD, coronary artery disease; HF, heart failure; MM, multiple myeloma; ACE-I, angiotensin converting enzyme inhibitors; ARB, angiotensin receptor blockers; ARNI, angiotensin receptor neprilysin inhibitor; CAD, coronary artery disease; EMB, endomyocardial biopsy; PYP, Technetium Pyrophosphate Scintigraphy; CMR, cardiovascular magnetic resonance; LVEDVI, left ventricular end diastolic volume index; LVEF, left ventricular ejection fraction; ECV, extracellular volume fraction; LGE, late gadolinium enhancement imaging; and CA, cardiac amyloidosis.

**Table S3.** Detailed Baseline (Table S3A) and CMR (Table S3B) Characteristics of Patients with TTR Amyloidosis.

| **A.** Baseline Characteristics. | | | | | | | | | | | | | | | | | | | | | | | | |
| --- | --- | --- | --- | --- | --- | --- | --- | --- | --- | --- | --- | --- | --- | --- | --- | --- | --- | --- | --- | --- | --- | --- | --- | --- |
| Pt# | Gender (male – 1) | Race (AA – 1) | Age | | Death | | HTN (1-present; 2- poorly controlled; 3 – longstanding) | | HTN duration | | | DM | | 1 – CKD; 2- ESRD | | CAD | | HF | Atrial arrhythmia | | MM | | | ACE-I/ARB/ARNI |
| 1 | 1 | 1 | 61 | | 1 | | 1 | | At least 2 years | | | 0 | | 1 | | 1 | | 1 | 1 | | 0 | | | 1 |
| 2 | 1 | 1 | 74 | | 0 | | 1 | | At least 6 years | | | 1 | | 0 | | 0 | | 1 | 0 | | 0 | | | 1 |
| 3 | 1 | 0 | 82 | | 0 | | 1 | | At least 8 years | | | 0 | | 0 | | 1 | | 1 | 1 | | 0 | | | 1 |
| 4 | 1 | 1 | 71 | | 1 | | 1 | | At least 9 years | | | 0 | | 0 | | 1 | | 1 | 0 | | 0 | | | 1 |
| 5 | 1 | 1 | 64 | | 1 | | 1 | | At least 1 year | | | 0 | | 1 | | 0 | | 1 | 1 | | 0 | | | 1 |
| 6 | 1 | 1 | 71 | | 0 | | 1 | | At least 5 years | | | 0 | | 1, 2 | | 1 | | 1 | 1 | | 0 | | | 0 |
| 7 | 1 | 1 | 78 | | 1 | | 1 | | At least 7 years | | | 1 | | 1 | | 1 | | 1 | 0 | | 0 | | | 1 |
| 8 | 1 | 0 | 78 | | 0 | | 1 | | At least 6 years | | | 0 | | 0 | | 1 | | 1 | 0 | | 0 | | | 0 |
| 9 | 1 | 0 | 86 | | 1 | | 1 | | At least 4 years | | | 0 | | 1 | | 1 | | 1 | 1 | | 0 | | | 1 |
| 10 | 1 | 0 | 71 | | 1 | | 1 | | At least 6 years | | | 1 | | 1 | | 1 | | 1 | 1 | | 0 | | | 0 |
| 11 | 1 | 0 | 76 | | 1 | | 1 | | At least 2 years | | | 0 | | 0 | | 0 | | 1 | 1 | | 0 | | | 1 |
| 12 | 1 | 1 | 77 | | 1 | | 1 | | At least 10 years | | | 0 | | 0 | | 0 | | 1 | 0 | | 0 | | | 1 |
| 13 | 1 | 1 | 77 | | 0 | | 1 | | At least 1 year | | | 0 | | 1 | | 1 | | 1 | 1 | | 0 | | | 0 |
| 14 | 1 | 0 | 70 | | 0 | | 1 | | At least 7 years | | | 0 | | 1 | | 1 | | 1 | 1 | | 0 | | | 0 |
| 15 | 1 | 0 | 72 | | 1 | | 1 | | At least 1 year | | | 1 | | 1 | | 1 | | 1 | 1 | | 0 | | | 0 |
| 16 | 0 | 0 | 58 | | 1 | | 1 | | At least 4 years | | | 0 | | 0 | | 0 | | 1 | 1 | | 0 | | | 0 |
| 17 | 1 | 1 | 59 | | 0 | | 1 | | At least 8 years | | | 0 | | 1 | | 0 | | 1 | 0 | | 0 | | | 0 |
| **B.** Diagnostic characteristics. | | | | | | | | | | | | | | | | | | | | | | | | |
| Pt# | Ischemic evaluation (1- done)/obstructive CAD (1 – positive) | EMB result (1- myocyte hypertrophy; 2-inflammation; 3-interstitial fibrosis; 4-“+” CA stains); 5 – EMB performed twice/  EMB date | | PYP scan (1- done, 2 – negative) | | Genetic testing (1 - done, 2 – positive) | | CMR result/  CMR date | | Max  thickness  (cm) | LVEDVI (ml/m2) | | LVEF (%) | | ECV | | LGE in the PM | | | Number of segments  with subendocardial LGE | | Total number of LGE segments | LGE extent | |
| 1 | 1/1 | 4/  2016 | | 0 | | 1, 2 | | Suggestive of CA/  2016 | | 2.2 | 70 | | 44 | | 37.8 | | 1 | | | 4 | | 8 | 40.7 | |
| 2 | 0 | 4/  2016 | | 1, 2 | | 1, 2 | | Suggestive of CA/  2016 | | 2.4 | 87 | | 48 | | 55.8 | | 1 | | | 12 | | 17 | 30.8 | |
| 3 | 1/1 | 4/  2020 | | 1 | | 1 | | Suggestive of CA/  2020 | | 1.6 | 70 | | 59 | | 42.1 | | 1 | | | 0 | | 4 | 41.8 | |
| 4 | 1/1 | 4/  2019 | | 1 | | 1,2 | | Suggestive of CA/  2019 | | 2.0 | 77 | | 39 | | 67.2 | | 1 | | | 3 | | 17 | 47.2 | |
| 5 | 1/0 | 4/  2014 | | 0 | | 1, 2 | | Suggestive of CA/  2013 | | 2.0 | 122 | | 29 | | 58.1 | | 1 | | | 0 | | 17 | 43.3 | |
| 6 | 1/0 | 4/  2012 | | 0 | | 1, 2 | | Suggestive of CA/  2012 | | 2.5 | 110 | | 25 | | 74.6 | | 1 | | | 0 | | 17 | 40.8 | |
| 7 | 1/0 | 4/  2017 | | 0 | | 1, 2 | | Suggestive of CA/  2017 | | 2.3 | 92 | | 30 | |  | | 1 | | | 0 | | 15 | 44.3 | |
| 8 | 1/0 | 4/  2017 | | 0 | | 0 | | Suggestive of NICM/  2017 | | 1.3 | 46 | | 61 | | 35.5 | | 0 | | | 2 | | 6 | 26.5 | |
| 9 | 1/1 | 4/  2016 | | 0 | | 1 | | Suggestive of CA/  2016 | | 1.9 | 98 | | 48 | | 46.3 | | 0 | | | 0 | | 12 | 7.6 | |
| 10 | 1/1 | 4/  2012 | | 0 | | 1 | | Suggestive of CA/  2012 | | 2.0 | 107 | | 30 | | 54.3 | | 1 | | | 2 | | 17 | 32.3 | |
| 11 | 1/0 | 4/  2013 | | 0 | | 1, 2 | | Suggestive of CA  /2013 | | 2.1 | 86 | | 29 | | 65.6 | | 1 | | | 0 | | 17 | 49.3 | |
| 12 | 1/0 | 4/  2013 | | 0 | | 0 | | Suggestive of CA/  2013 | | 1.9 | 77 | | 46 | | 44.4 | | 1 | | | 0 | | 13 | 49.5 | |
| 13 | 1/0 | 4/  2012 | | 0 | | 0 | | Suggestive of CA/  2012 | | 2.1 | 82 | | 44 | | 62.3 | | 1 | | | 0 | | 13 | 53.4 | |
| 14 | 1/0 | 4/  2021 | | 1 | | 1 | | Suggestive of CA/  2020 | | 2.5 | 122 | | 45 | | 63.7 | | 1 | | | 4 | | 17 | 44.1 | |
| 15 | 1/1 | 4/  2015 | | 0 | | 1 | | Suggestive of CA/  2015 | | 1.8 | 82 | | 30 | | 68.4 | | 1 | | | 2 | | 17 | 32.3 | |
| 16 | 1/0 | 4/  2015 | | 0 | | 0 | | Suggestive of CA/  2015 | | 1.9 | 76 | | 37 | | 79.7 | | 1 | | | 0 | | 17 | 37.3 | |
| 17 | 1/0 | 4/  2018 | | 1 | | 1, 2 | | Suggestive of CA/  2020 | | 2.4 | 92 | | 35 | | 89.2 | | 1 | | | 0 | | 17 | 55.8 | |

TTR indicates transthyretin; AA, African American; HTN, hypertension; DM, diabetes mellitus; CKD, chronic kidney disease; CAD, coronary artery disease; HF, heart failure; MM, multiple myeloma; ACE-I, angiotensin converting enzyme inhibitors; ARB, angiotensin receptor blockers; ARNI, angiotensin receptor neprilysin inhibitor; CAD, coronary artery disease; EMB, endomyocardial biopsy; PYP, Technetium Pyrophosphate Scintigraphy; CMR, cardiovascular magnetic resonance; LVEDVI, left ventricular end diastolic volume index; LVEF, left ventricular ejection fraction; ECV, extracellular volume fraction; LGE, late gadolinium enhancement imaging; CA, cardiac amyloidosis; and NICM, nonischemic cardiomyopath


**Table S4**. Intraobserver and Interobserver Reproducibility of T1 Mapping, ECV, and Selected LGE Parameters.

| **Categorical Variable** | **Intraobserver reproducibility** | | | | | | **Interobserver reproducibility** | | | | | |
| --- | --- | --- | --- | --- | --- | --- | --- | --- | --- | --- | --- | --- |
|  | Proportion in agreement | | Kappa  95% CI | | p | | Proportion in agreement | | Kappa  95% CI | | p | |
| LGE in papillary muscles | 90% | | 0.80 | | 0.01 | | 100% | | 1 | | 0.002 | |
| **Continuous Variables** | **Intraobserver reproducibility** | | | | | | **Interobserver reproducibility** | | | | | |
|  | Mean Difference | Limits of agreement | | Error range | | ICC | Mean Difference | Limits of agreement | | Error range | | ICC |
| Pre-contrast myocardial T1 mapping (ms) | 4.2 | -23.5 to 31.9 | | 19.6 | | 0.97  (0.88-0.99) | 1.4 | -27.8 to 30.6 | | 20.6 | | 0.97  (0.87-0.99) |
| Post-contrast myocardial T1 mapping (ms) | 1.7 | -9.5 to 12.9 | | 7.94 | | 0.99  (0.98-0.99) | -3.9 | -35.0 to 27.2 | | 22.0 | | 0.98  (0.91-0.99 |
| ECV % | -0.16 | -2.6 to 2.3 | | 1.70 | | 0.99  (0.99-1.0) | -0.22 | -5.4 to 5.0 | | 3.67 | | 0.99  (0.96-0.99) |
| AHA segments with LGE | 0.1 | -0.5 to 0.7 | | 0.44 | | 0.99  (0.99-1.0) | -0.1 | -1.8 to 1.6 | | 1.21 | | 0.99  (0.97-0.99) |
| AHA segments with subendocardial LGE | 0.2 | -0.6 to 1.0 | | 0.58 | | 0.99  (0.99-1.0) | 0.4 | -0.6 to 1.4 | | 0.72 | | 0.99  (0.97-1.0) |
| AHA segments with midmyocardial LGE | 0 | -0.9 to 0.9 | | 0.65 | | 0.98  (0.95-0.99) | -0.4 | -2.5 to 1.7 | | 1.49 | | 0.93  (0.74-0.98) |
| AHA segments with transmural LGE | -0.1 | -0.7 to 0.5 | | 0.44 | | 0.99  (0.99-1.0) | -0.1 | -0.7 to 0.5 | | 0.44 | | 0.99  (0.99-1.0) |
| AHA segments with subendocardial and transmural LGE | 0.1 | -0.5 to 0.7 | | 0.44 | | 0.99  (0.99- 1.0) | 0.3 | -0.7 to 1.3 | | 0.67 | | 0.99  (0.99- 1.0) |
| LGE extent (%) | -0.4 | -3.1 to 2.3 | | 1.89 | | 0.99  (0.99-0.99) | -0.5 | -5.8 to 4.9 | | 3.78 | | 0.99  (0.97-0.99) |
| Query Score Total | -0.1 | -0.72 to 0.52 | | 0.44 | | 0.99  (0.99-1.0) | 0.3 | -3.84 to 4.44 | | 2.93 | | 0.99  (0.99-1.0) |

ECV indicates extracellular volume fraction; LGE, late gadolinium enhancement; and AHA, American Heart Association.

**Figure S1.** Clinical Decision Making in Patients with Hypertension, Left Ventricular Hypertrophy, and Heart Failure.

**
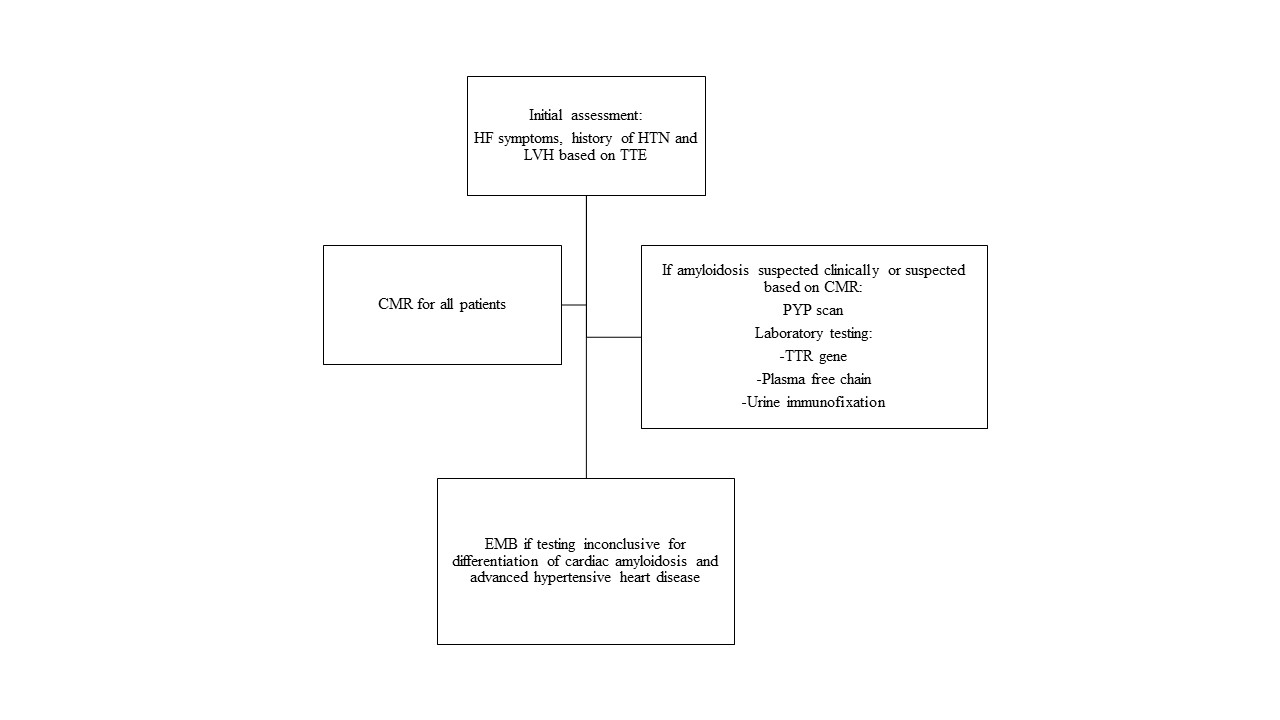
**

HTN indicates hypertension; LVH, left ventricular hypertrophy; HF, heart failure; TTE, transthoracic echocardiography; CMR, cardiovascular magnetic resonance; PYP; EMB, endomyocardial biopsy.

**Figure S2.** CMR Parameters in Study Groups Defined by the Final Diagnosis of Hypertensive Cardiomyopathy, AL Amyloidosis, and ATTR Amyloidosis.

**
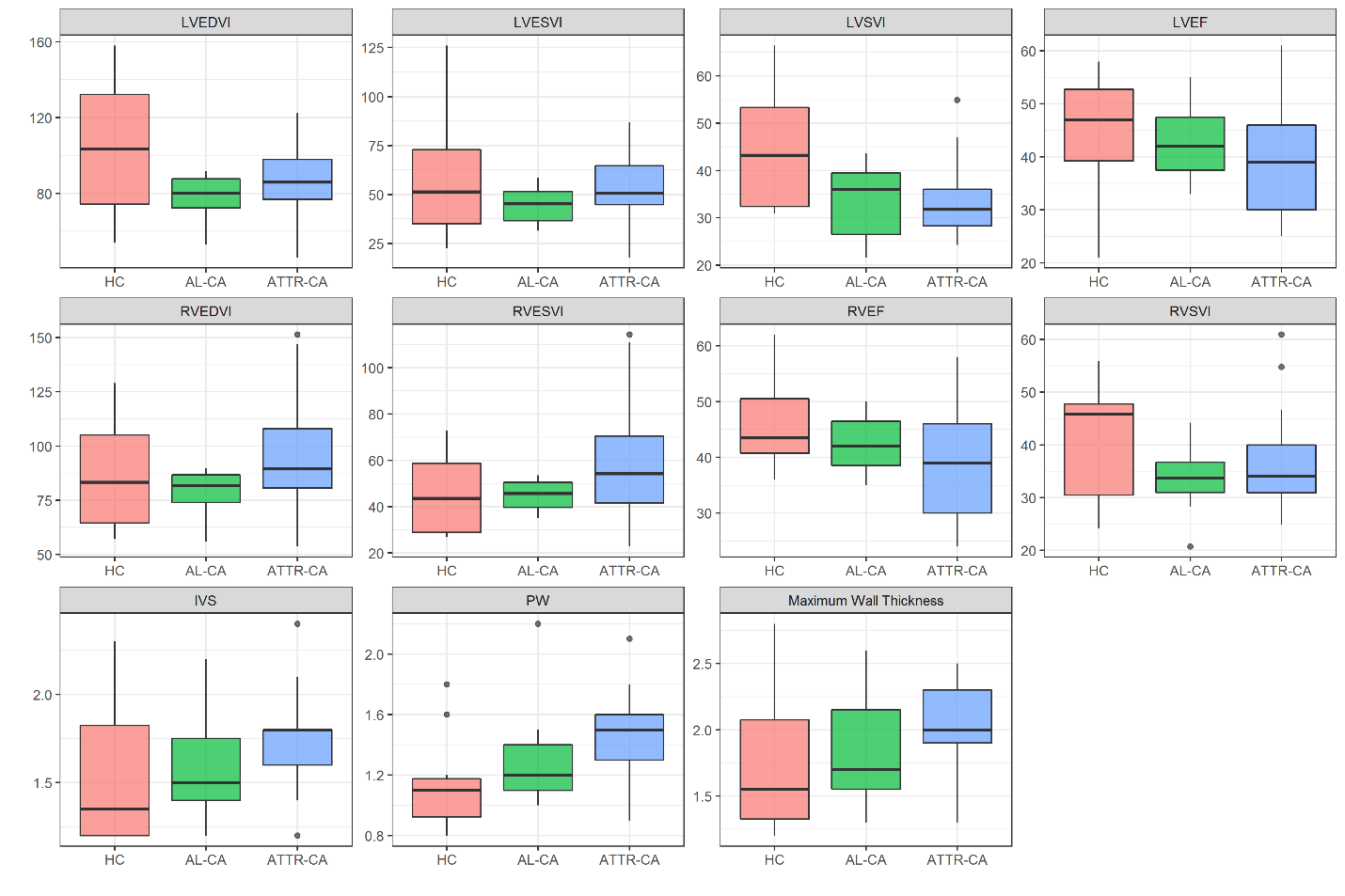
**

HC indicates hypertensive cardiomyopathy; AL-CA, light chain cardiac amyloidosis; ATTR-CA, transthyretin cardiac amyloidosis; LVEDVI, left ventricular end-diastolic volume index; LVESVI, left ventricular end-systolic volume index; LVSVI, left ventricular stroke volume index; LVEF, left ventricular ejection fraction; RVEDVI, right ventricular end-diastolic volume index; RVESVI, right ventricular end-systolic volume index; RVEF, right ventricular ejection fraction; RVSVI, right ventricular stroke volume index; IVS, interventricular septum; and PW, posterior wall.

**Figure S3.** LGE Patterns in Study Groups Defined by the Final Diagnosis of Hypertensive Cardiomyopathy, AL Amyloidosis, and ATTR Amyloidosis.

**
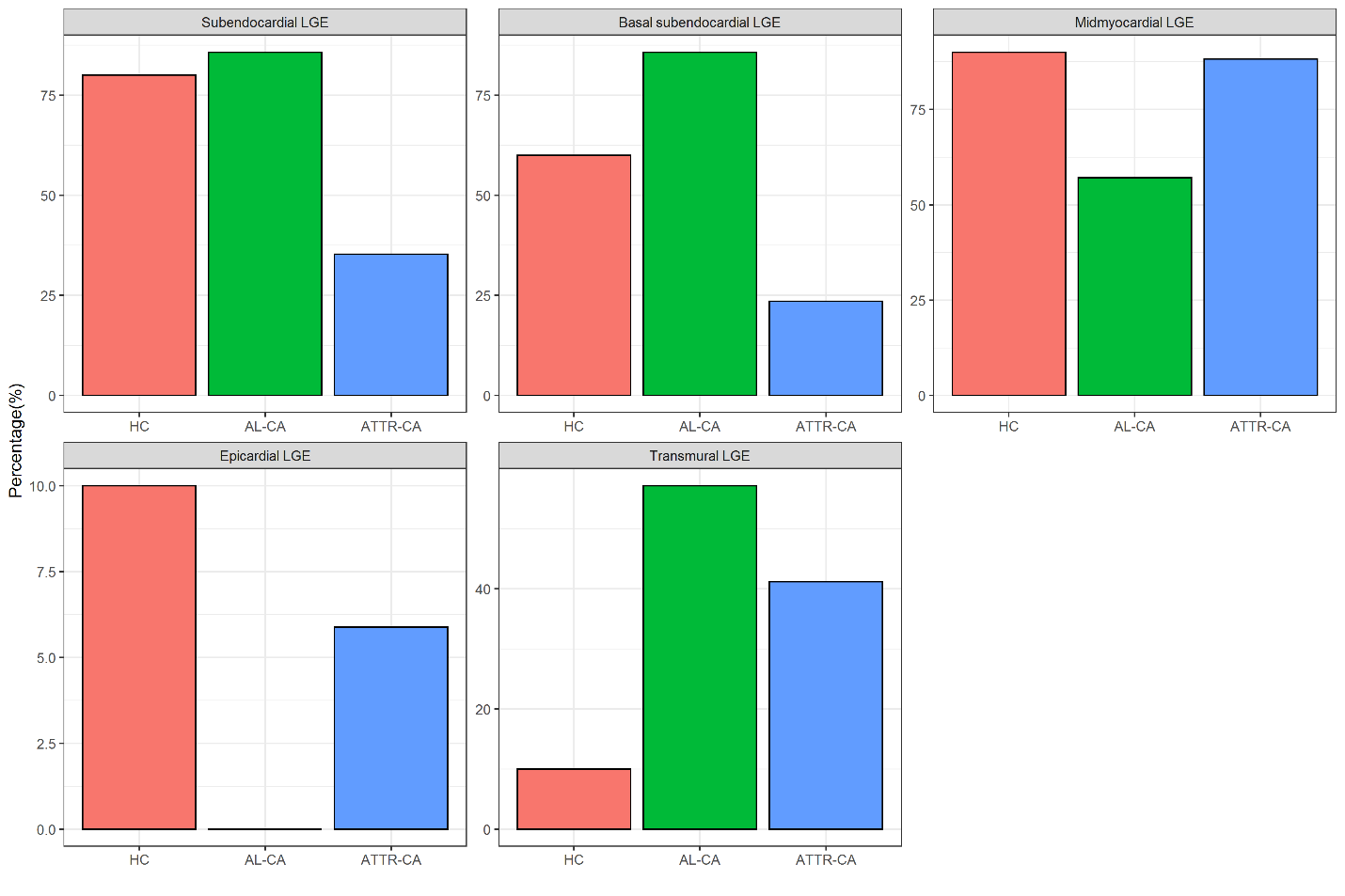
**

LGE indicates late gadolinium enhancement imaging; HC, hypertensive cardiomyopathy; AL CA, light chain cardiac amyloidosis; ATTR CA, transthyretin cardiac amyloidosis.

**Figure S4.** AHA Segments with LGE in Study Groups Defined by the Final Diagnosis of Hypertensive Cardiomyopathy, AL Amyloidosis, and ATTR Amyloidosis.

**
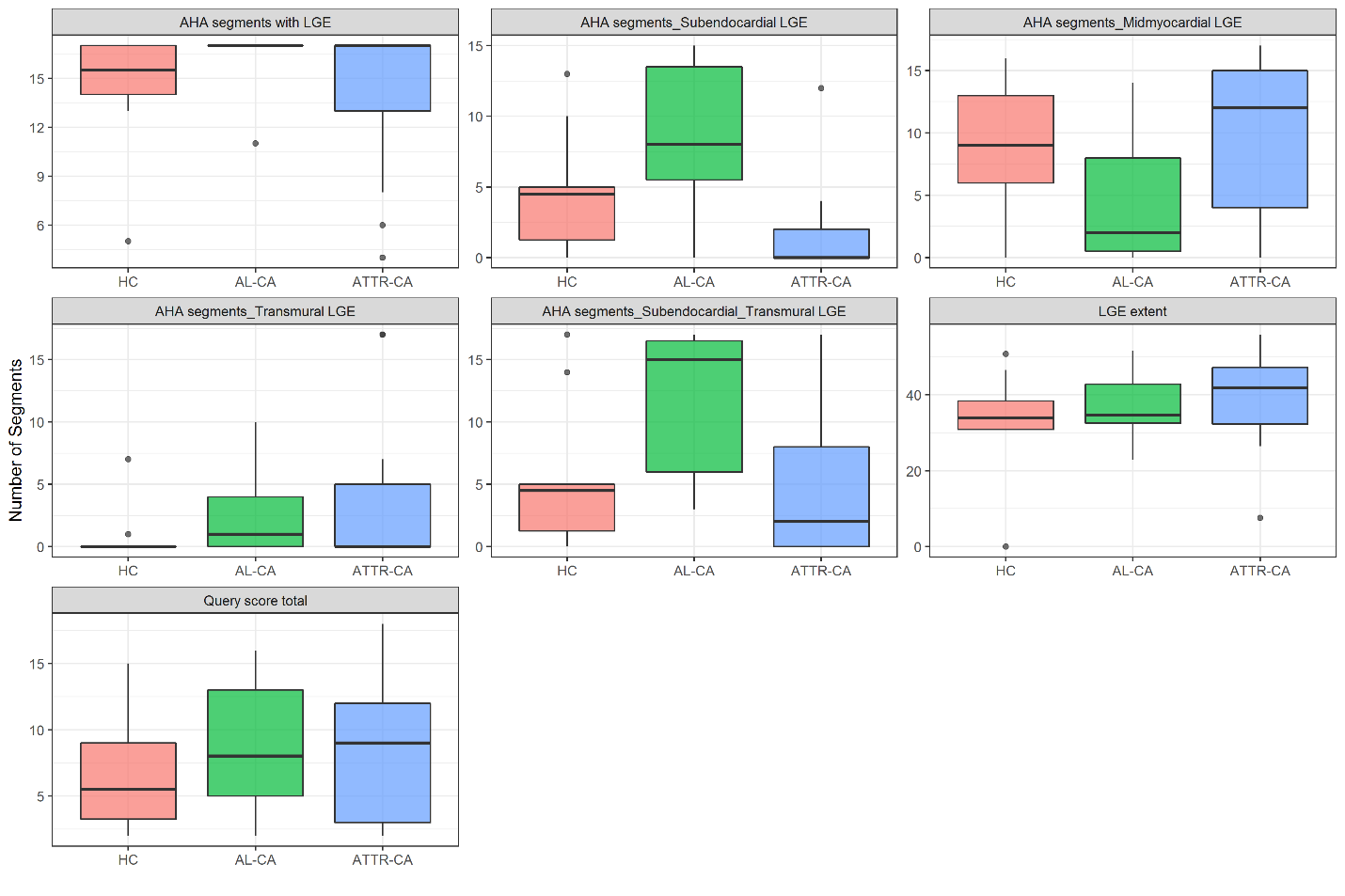
**

AHA indicates American Heart Association; LGE, late gadolinium enhancement imaging; HHD, hypertensive cardiomyopathy; AL CA, light chain cardiac amyloidosis; ATTR CA, transthyretin cardiac amyloidosis.
